# Diaqua­bis­(5-carb­oxy-2-propyl-1*H*-imidazole-4-carboxyl­ato-κ^2^ *N* ^3^,*O* ^4^)cobalt(II) 3.5-hydrate

**DOI:** 10.1107/S1600536810052785

**Published:** 2010-12-24

**Authors:** Shi-Jie Li, Dong-Liang Miao, Wen-Dong Song, Shao-Wei Tong, Jing-Bo An

**Affiliations:** a College of Food Science and Technology, Guangdong Ocean University, Zhanjiang 524088, People’s Republic of China; b College of Science, Guangdong Ocean University, Zhanjiang 524088, People’s Republic of China

## Abstract

In the title complex, [Co(C_8_H_9_N_2_O_4_)_2_(H_2_O)_2_]·3.5H_2_O, the Co^II^ cation is six-coordinated by two H_2_pimda^−^ ligands (H_3_pimda is 2-propyl-1*H*-imidazole-4,5-carboxylic acid) and two water mol­ecules in a distorted octa­hedral environment. The crystal structures features a three-dimensional network stabilized by extensive O—H⋯O and N—H⋯O hydrogen bonds. The propyl groups of the ligands are disordered over two sets of sites with refined occupancies of 0.673 (8):0.327 (8) and 0.621 (17):0.379 (17). One of the water mol­ecules is located on a site with half-occupancy.

## Related literature

For our past work based on H_3_pimda, see: Yan *et al.* (2010 ▶); Li, Dong *et al.* (2010 ▶); Song *et al.* (2010 ▶); He *et al.* (2010 ▶); Fan *et al.* (2010 ▶); Li, Miao *et al.* (2010 ▶); Li, Song *et al.* (2010 ▶).
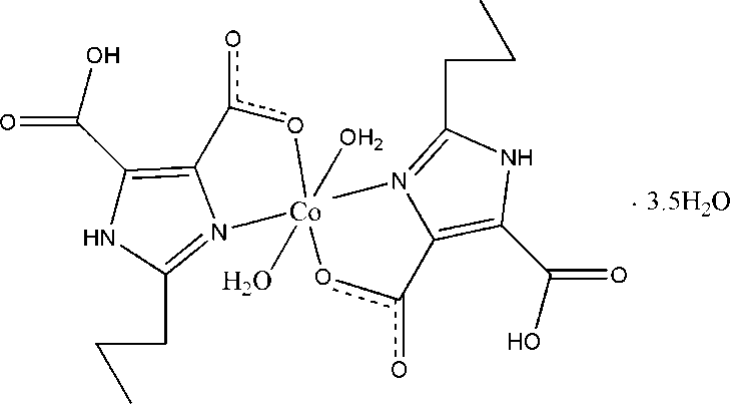


## Experimental

### 

#### Crystal data

[Co(C_8_H_9_N_2_O_4_)_2_(H_2_O)_2_]·3.5H_2_O
*M* *_r_* = 552.36
Triclinic, 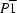

*a* = 10.405 (1) Å
*b* = 10.6131 (11) Å
*c* = 11.2529 (13) Å
α = 82.371 (1)°
β = 83.743 (1)°
γ = 87.330 (2)°
*V* = 1223.7 (2) Å^3^
*Z* = 2
Mo *K*α radiation
μ = 0.77 mm^−1^
*T* = 298 K
0.18 × 0.09 × 0.07 mm

#### Data collection

Bruker SMART 1000 CCD area-detector diffractometer
Absorption correction: multi-scan (*SADABS*; Bruker, 2004 ▶) *T*_min_ = 0.873, *T*_max_ = 0.948
6529 measured reflections
4249 independent reflections
2522 reflections with *I* > 2σ(*I*)
*R*_int_ = 0.034

#### Refinement

*R*[*F* ^2^ > 2σ(*F* ^2^)] = 0.050
*wR*(*F* ^2^) = 0.101
*S* = 1.03
4249 reflections
376 parameters
18 restraints
H-atom parameters constrained
Δρ_max_ = 0.33 e Å^−3^
Δρ_min_ = −0.31 e Å^−3^

### 

Data collection: *SMART* (Bruker, 2004 ▶); cell refinement: *SAINT* (Bruker, 2004 ▶); data reduction: *SAINT*; program(s) used to solve structure: *SHELXS97* (Sheldrick, 2008 ▶); program(s) used to refine structure: *SHELXL97* (Sheldrick, 2008 ▶); molecular graphics: *SHELXTL* (Sheldrick, 2008 ▶); software used to prepare material for publication: *SHELXTL*.

## Supplementary Material

Crystal structure: contains datablocks I, global. DOI: 10.1107/S1600536810052785/bt5437sup1.cif

Structure factors: contains datablocks I. DOI: 10.1107/S1600536810052785/bt5437Isup2.hkl

Additional supplementary materials:  crystallographic information; 3D view; checkCIF report

## Figures and Tables

**Table 1:** Hydrogen-bond geometry (Å, °)

*D*—H⋯*A*	*D*—H	H⋯*A*	*D*⋯*A*	*D*—H⋯*A*
N2—H2⋯O4*W*	0.86	1.89	2.745 (5)	171
N4—H4⋯O5*W*^i^	0.86	1.93	2.752 (5)	160
O3—H3⋯O2	0.82	1.68	2.500 (4)	179
O7—H7⋯O6	0.82	1.64	2.461 (4)	176
O1*W*—H1*W*⋯O8^ii^	0.85	1.87	2.715 (4)	178
O1*W*—H2*W*⋯O3*W*^iii^	0.85	1.81	2.661 (4)	177
O2*W*—H4*W*⋯O7*W*^iv^	0.85	1.94	2.791 (4)	174
O2*W*—H3*W*⋯O8^v^	0.85	2.05	2.897 (4)	175
O3*W*—H5*W*⋯O2^iv^	0.85	1.95	2.796 (5)	172
O3*W*—H6*W*⋯O5^vi^	0.85	2.05	2.895 (4)	172
O3*W*—H6*W*⋯O6^vi^	0.85	2.63	3.206 (4)	127
O4*W*—H8*W*⋯O6*W*	0.85	1.89	2.674 (7)	152
O5*W*—H9*W*⋯O3*W*^iii^	0.85	2.08	2.867 (5)	153
O5*W*—H10*W*⋯O7*W*^iv^	0.85	2.33	3.092 (5)	149
O6*W*—H12*W*⋯O6*W*^vii^	0.85	1.68	2.162 (11)	113
O6*W*—H12*W*⋯O1^viii^	0.85	2.14	2.730 (6)	126
O6*W*—H11*W*⋯O5*W*^iv^	0.85	2.05	2.588 (7)	121

Symmetry codes: (i) 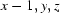; (ii) 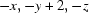; (iii) 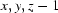; (iv) 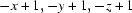; (v) 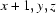; (vi) 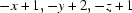; (vii) 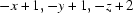; (viii) 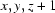.

**Fig. 1.**
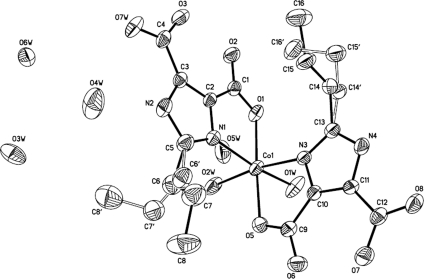
The structure of the title compound, showing the atomic numbering scheme. Non-H atoms are shown with 30% probability displacement ellipsoids.

## supplementary crystallographic information

### Comment

The 2-propyl-1*H*-imidazole-4,5-carboxylate (H_3_pimda) ligand has been used to obtain new metal-organic complexes by our research group, such as poly[diaquabis(5-carboxy-2-propyl-1*H*-imidazole-4-carboxylato-k^3^ N^3^, O^4^,*O*^5^)calcium(II)] (Song *et al.*, 2010), [diaquabis(5-carboxy-2-propyl-1*H*-imidazole-4-carboxylato-k^2^N^3^,\ *O*^4^) manganese(II)]*N*,*N*-dimethylformamide (Yan *et al.*, 2010), Diaquabis(4-carboxy-2-propyl-1*H*-imidazole-5-carboxylato- k^2^N^3^,*O*^4^)copper(II) *N*,*N*-dimethylformamide disolvate (He *et al.*, 2010), Diaquabis(5-carboxy-2-propyl-1*H*-imidazole- 4-carboxylato-k^2^N^3^,*O*^4^)nickel(II) tetrahedrate (Fan *et al.*, 2010), Diaquabis(5-carboxy-2-propyl-1*H*-imidazole-4-carboxylato- k^2^N^3^,*O*^4^)-manganese(II) 3.5-hydrate (Li, Miao *et al.*, 2010), Diaquabis (5-carboxy-2-propyl-1*H*-imidazole-4-carboxylato-K^2^N^3^,*O*^4^)\ zinc(II) 3.5-hydrate (Li, Song *et al.* 2010) and Diaquabis(5-carboxy-2-propyl-1*H*- imidazole-4-carboxylato-k^2^N^3^,*O*^4^)cadmium(II) 3.5-hydrate (Li, Dong *et al.* 2010). In this paper, we report the synthesis and structure of a new Co(II) complex based the same ligand.

As illustrated in figure 1, the title complex molecule is isomorphous with Ni(II), Mn(II), Cd(II) and Zn(II) analogues (Fan *et al.*, 2010; Li, Dong *et al.*, 2010; Li, Song *et al.*, 2010; Li, Miao *et al.*, 2010), Similar structural description applies to the present isomorphous complex. The Co^II^ is six-coordinated in a distorted octahedral geometry. the H_3_pimda acts as a bidentate mode to chelate the center Co(II). one carboxy group of the ligand was delocalized and the other was protonated, indicated by the difference of the bond lengths. The dihedral angle between the two imidazole rings is 84.2 (2) %A. In the crystal structure, the three-dimensional supramolecular framework is stabilized by extensive O—H···O and N—H···O hydrogen bonds.The propyl groups of H_3_pimda are disordered over two sets of sites with refined occupiencies of 0.673 (8):0.327 (8) and 0.621 (17): 0.379 (17). One of the water molecules is half occupied.

### Experimental

A mixture of Co(NO_3_)_2_ (0.5 mmol, 0.06 g) and 2-propyl-1*H*-imidazole-4,5-dicarboxylic acid(0.5 mmol, 0.99 g) in 15 ml of H_2_O solution was sealed in an autoclave equipped with a Teflon liner (20 ml) and then heated at 433K for 4 days. Crystals of the title compound were obtained by slow evaporation of the solvent at room temperature.

### Refinement

Water H atoms were located in a difference Fourier map and were allowed to ride on the parent atom, with *U*_iso_(H) = 1.5*U*_eq_(O). Carboxyl H atoms were located in a difference map and refined with distance restraints, *U*_iso_(H) = 1.5*U*_eq_(O). Other H atoms were placed at calculated positions and were treated as riding on parent atoms with C—H = 0.96 (methyl), 0.97 (methylene) and N—H = 0.86 Å, *U*_iso_(H) = 1.2 or 1.5*U*_eq_(C,N). The propyl groups of H_3_pimda are disordered over two sites with refined occupancies of 0.673 (8):0.327 (8) and 0.621 (17):0.379 (17). C—C distance restraints  were applied for the disordered components. The O3W water molecule is located close to an inversion centre, its occupancy factor was refined to 0.49 (1) and was fixed as 0.5 at the final refinements.

### Crystal data

**Table:**

[Co(C_8_H_9_N_2_O_4_)_2_(H_2_O)_2_]·3.5H_2_O	*Z* = 2
*M**_r_* = 552.36	*F*(000) = 576
Triclinic, *P*1	*D*_x_ = 1.499 Mg m^−^^3^
Hall symbol: -P 1	Mo *K*α radiation, λ = 0.71073 Å
*a* = 10.405 (1) Å	Cell parameters from 1702 reflections
*b* = 10.6131 (11) Å	θ = 2.5–25.9°
*c* = 11.2529 (13) Å	µ = 0.77 mm^−^^1^
α = 82.371 (1)°	*T* = 298 K
β = 83.743 (1)°	Cube, red
γ = 87.330 (2)°	0.18 × 0.09 × 0.07 mm
*V* = 1223.7 (2) Å^3^	

### Data collection

**Table:**

Bruker SMART 1000 CCD area-detector diffractometer	4249 independent reflections
Radiation source: fine-focus sealed tube	2522 reflections with *I* > 2σ(*I*)
graphite	*R*_int_ = 0.034
φ and ω scans	θ_max_ = 25.0°, θ_min_ = 1.8°
Absorption correction: multi-scan (*SADABS*; Bruker, 2004)	*h* = −12→11
*T*_min_ = 0.873, *T*_max_ = 0.948	*k* = −12→11
6529 measured reflections	*l* = −13→12

### Refinement

**Table:**

Refinement on *F*^2^	Primary atom site location: structure-invariant direct methods
Least-squares matrix: full	Secondary atom site location: difference Fourier map
*R*[*F*^2^ > 2σ(*F*^2^)] = 0.050	Hydrogen site location: inferred from neighbouring sites
*wR*(*F*^2^) = 0.101	H-atom parameters constrained
*S* = 1.03	*w* = 1/[σ^2^(*F*_o_^2^) + (0.0305*P*)^2^] where *P* = (*F*_o_^2^ + 2*F*_c_^2^)/3
4249 reflections	(Δ/σ)_max_ = 0.001
376 parameters	Δρ_max_ = 0.33 e Å^−^^3^
18 restraints	Δρ_min_ = −0.31 e Å^−^^3^

### Special details

**Table:**

Geometry. All e.s.d.'s (except the e.s.d. in the dihedral angle between two l.s. planes) are estimated using the full covariance matrix. The cell e.s.d.'s are taken into account individually in the estimation of e.s.d.'s in distances, angles and torsion angles; correlations between e.s.d.'s in cell parameters are only used when they are defined by crystal symmetry. An approximate (isotropic) treatment of cell e.s.d.'s is used for estimating e.s.d.'s involving l.s. planes.
Refinement. Refinement of *F*^2^ against ALL reflections. The weighted *R*-factor *wR* and goodness of fit *S* are based on *F*^2^, conventional *R*-factors *R* are based on *F*, with *F* set to zero for negative *F*^2^. The threshold expression of *F*^2^ > σ(*F*^2^) is used only for calculating *R*-factors(gt) *etc*. and is not relevant to the choice of reflections for refinement. *R*-factors based on *F*^2^ are statistically about twice as large as those based on *F*, and *R*- factors based on ALL data will be even larger.

### Fractional atomic coordinates and isotropic or equivalent isotropic displacement parameters (Å^2^)

**Table:**

	*x*	*y*	*z*	*U*_iso_*/*U*_eq_	Occ. (<1)
Co1	0.34903 (6)	0.79029 (6)	0.19293 (6)	0.0468 (2)	
N1	0.3265 (3)	0.7131 (3)	0.3764 (3)	0.0447 (9)	
N2	0.3046 (3)	0.6416 (4)	0.5685 (3)	0.0548 (11)	
H2	0.2910	0.6408	0.6453	0.066*	
N3	0.1480 (3)	0.7861 (3)	0.1738 (3)	0.0419 (9)	
N4	−0.0526 (3)	0.7781 (4)	0.1332 (3)	0.0504 (10)	
H4	−0.1231	0.7478	0.1171	0.060*	
O1	0.3958 (3)	0.5942 (3)	0.1833 (3)	0.0525 (8)	
O2	0.4033 (3)	0.4039 (3)	0.2939 (3)	0.0629 (9)	
O3	0.3743 (3)	0.3174 (3)	0.5125 (3)	0.0670 (10)	
H3	0.3830	0.3458	0.4408	0.100*	
O7W	0.3316 (3)	0.3924 (3)	0.6857 (3)	0.0687 (11)	
O5	0.2850 (3)	0.9854 (3)	0.2050 (3)	0.0533 (9)	
O6	0.1184 (3)	1.1214 (3)	0.1846 (3)	0.0633 (10)	
O7	−0.1073 (3)	1.1143 (3)	0.1400 (3)	0.0606 (9)	
H7	−0.0327	1.1136	0.1571	0.091*	
O8	−0.2424 (3)	0.9744 (3)	0.1005 (3)	0.0585 (9)	
O1W	0.3850 (3)	0.8336 (3)	0.0107 (3)	0.0766 (12)	
H1W	0.3418	0.8937	−0.0259	0.115*	
H2W	0.4514	0.8160	−0.0359	0.115*	
O2W	0.5401 (3)	0.8259 (3)	0.2132 (3)	0.0758 (12)	
H4W	0.5741	0.7575	0.2459	0.114*	
H3W	0.6004	0.8732	0.1786	0.114*	
O3W	0.5950 (3)	0.7723 (3)	0.8703 (3)	0.0881 (12)	
H5W	0.5992	0.7238	0.8155	0.132*	
H6W	0.6319	0.8410	0.8414	0.132*	
O4W	0.2706 (4)	0.6669 (4)	0.8096 (3)	0.1170 (16)	
H7W	0.2264	0.7287	0.8359	0.176*	
H8W	0.2895	0.6117	0.8675	0.176*	
O5W	0.7616 (4)	0.6532 (4)	0.0420 (4)	0.1161 (15)	
H9W	0.7321	0.7065	−0.0124	0.174*	
H10W	0.7077	0.6476	0.1048	0.174*	
O6W	0.4094 (5)	0.5155 (6)	0.9607 (5)	0.0591 (18)	0.50
H12W	0.4516	0.5578	1.0016	0.089*	0.50
H11W	0.3534	0.4759	1.0109	0.089*	0.50
C1	0.3847 (4)	0.5225 (5)	0.2824 (4)	0.0453 (12)	
C2	0.3472 (4)	0.5846 (4)	0.3905 (4)	0.0388 (11)	
C3	0.3330 (4)	0.5379 (4)	0.5099 (4)	0.0424 (11)	
C4	0.3452 (4)	0.4105 (5)	0.5768 (5)	0.0503 (13)	
C5	0.3013 (5)	0.7456 (5)	0.4860 (5)	0.0585 (14)	
C6	0.2961 (17)	0.880 (3)	0.514 (2)	0.073 (6)	0.673 (8)
H6A	0.3453	0.9326	0.4496	0.088*	0.673 (8)
H6B	0.3353	0.8828	0.5880	0.088*	0.673 (8)
C7	0.1592 (11)	0.9306 (11)	0.5282 (9)	0.090 (3)	0.673 (8)
H7A	0.1126	0.8861	0.5997	0.108*	0.673 (8)
H7B	0.1159	0.9180	0.4589	0.108*	0.673 (8)
C8	0.1612 (10)	1.0747 (8)	0.5395 (9)	0.128 (5)	0.673 (8)
H8A	0.2085	1.0867	0.6055	0.192*	0.673 (8)
H8B	0.0742	1.1075	0.5537	0.192*	0.673 (8)
H8C	0.2023	1.1188	0.4662	0.192*	0.673 (8)
C9	0.1692 (5)	1.0100 (5)	0.1877 (4)	0.0476 (12)	
C10	0.0912 (4)	0.9051 (4)	0.1699 (4)	0.0406 (11)	
C11	−0.0345 (4)	0.9016 (4)	0.1443 (4)	0.0410 (11)	
C12	−0.1370 (5)	1.0008 (5)	0.1268 (4)	0.0485 (13)	
C13	0.0583 (4)	0.7107 (5)	0.1515 (4)	0.0478 (12)	
C14	0.064 (3)	0.569 (3)	0.1696 (16)	0.054 (5)	0.621 (17)
H14A	0.1520	0.5390	0.1479	0.064*	0.621 (17)
H14B	0.0087	0.5375	0.1170	0.064*	0.621 (17)
C15	0.022 (2)	0.517 (2)	0.2987 (18)	0.068 (6)	0.621 (17)
H15A	−0.0693	0.5370	0.3172	0.081*	0.621 (17)
H15B	0.0697	0.5567	0.3521	0.081*	0.621 (17)
C16	0.0448 (10)	0.3732 (14)	0.3208 (11)	0.099 (5)	0.621 (17)
H16A	0.0004	0.3336	0.2659	0.148*	0.621 (17)
H16B	0.0127	0.3423	0.4021	0.148*	0.621 (17)
H16C	0.1359	0.3532	0.3083	0.148*	0.621 (17)
C6'	0.237 (3)	0.867 (5)	0.522 (4)	0.067 (11)	0.327 (8)
H6'1	0.1850	0.8512	0.5990	0.080*	0.327 (8)
H6'2	0.1827	0.9061	0.4619	0.080*	0.327 (8)
C7'	0.350 (2)	0.954 (2)	0.532 (2)	0.083 (7)	0.327 (8)
H7'1	0.4079	0.9571	0.4579	0.099*	0.327 (8)
H7'2	0.3148	1.0395	0.5379	0.099*	0.327 (8)
C8'	0.426 (2)	0.9112 (19)	0.637 (2)	0.118 (9)	0.327 (8)
H8'1	0.3673	0.8984	0.7099	0.177*	0.327 (8)
H8'2	0.4856	0.9752	0.6444	0.177*	0.327 (8)
H8'3	0.4721	0.8329	0.6252	0.177*	0.327 (8)
C14'	0.083 (5)	0.576 (5)	0.122 (3)	0.055 (8)	0.379 (17)
H14C	0.1738	0.5519	0.1256	0.066*	0.379 (17)
H14D	0.0623	0.5708	0.0407	0.066*	0.379 (17)
C15'	−0.0004 (18)	0.4828 (17)	0.212 (2)	0.068 (6)	0.379 (17)
H15C	−0.0910	0.5058	0.2064	0.082*	0.379 (17)
H15D	0.0148	0.3974	0.1907	0.082*	0.379 (17)
C16'	0.029 (4)	0.484 (4)	0.342 (3)	0.083 (12)	0.379 (17)
H16D	0.0090	0.4031	0.3881	0.125*	0.379 (17)
H16E	−0.0218	0.5500	0.3768	0.125*	0.379 (17)
H16F	0.1194	0.4990	0.3429	0.125*	0.379 (17)

### Atomic displacement parameters (Å^2^)

**Table:**

	*U*^11^	*U*^22^	*U*^33^	*U*^12^	*U*^13^	*U*^23^
Co1	0.0410 (4)	0.0498 (4)	0.0465 (4)	0.0032 (3)	−0.0074 (3)	0.0067 (3)
N1	0.047 (2)	0.045 (2)	0.042 (2)	0.0015 (18)	−0.0092 (18)	−0.001 (2)
N2	0.063 (3)	0.062 (3)	0.037 (2)	0.011 (2)	−0.0069 (19)	−0.002 (2)
N3	0.039 (2)	0.038 (2)	0.047 (2)	0.0006 (18)	−0.0086 (18)	0.0025 (18)
N4	0.039 (2)	0.054 (3)	0.057 (3)	−0.002 (2)	−0.0136 (19)	0.005 (2)
O1	0.058 (2)	0.059 (2)	0.0373 (19)	0.0130 (16)	−0.0062 (15)	0.0021 (16)
O2	0.090 (3)	0.046 (2)	0.053 (2)	0.0097 (19)	−0.0150 (18)	−0.0059 (17)
O3	0.087 (3)	0.052 (2)	0.059 (2)	0.002 (2)	−0.0148 (19)	0.0103 (19)
O7W	0.066 (2)	0.086 (3)	0.045 (2)	0.0101 (19)	−0.0042 (18)	0.0201 (19)
O5	0.0476 (19)	0.049 (2)	0.064 (2)	−0.0059 (16)	−0.0147 (16)	−0.0017 (16)
O6	0.060 (2)	0.044 (2)	0.087 (3)	0.0017 (17)	−0.0118 (18)	−0.0077 (19)
O7	0.049 (2)	0.056 (2)	0.076 (3)	0.0130 (18)	−0.0102 (17)	−0.0079 (19)
O8	0.0412 (19)	0.063 (2)	0.067 (2)	0.0035 (17)	−0.0112 (17)	0.0136 (18)
O1W	0.061 (2)	0.099 (3)	0.054 (2)	0.0337 (19)	0.0069 (17)	0.026 (2)
O2W	0.044 (2)	0.081 (3)	0.092 (3)	−0.0128 (18)	−0.0180 (18)	0.040 (2)
O3W	0.080 (3)	0.082 (3)	0.104 (3)	−0.030 (2)	0.029 (2)	−0.042 (2)
O4W	0.156 (4)	0.133 (4)	0.067 (3)	0.057 (3)	−0.025 (3)	−0.041 (3)
O5W	0.105 (3)	0.131 (4)	0.129 (4)	0.012 (3)	−0.060 (3)	−0.041 (3)
O6W	0.066 (4)	0.061 (4)	0.052 (4)	−0.008 (3)	−0.009 (3)	−0.012 (3)
C1	0.041 (3)	0.047 (3)	0.048 (3)	0.003 (2)	−0.011 (2)	−0.002 (3)
C2	0.034 (2)	0.042 (3)	0.039 (3)	0.001 (2)	−0.007 (2)	0.001 (2)
C3	0.033 (2)	0.049 (3)	0.044 (3)	0.002 (2)	−0.007 (2)	0.000 (2)
C4	0.037 (3)	0.064 (4)	0.047 (3)	0.001 (2)	−0.010 (2)	0.007 (3)
C5	0.073 (4)	0.051 (4)	0.051 (3)	0.011 (3)	−0.010 (3)	−0.007 (3)
C6	0.089 (16)	0.069 (11)	0.062 (8)	0.002 (15)	−0.006 (11)	−0.008 (7)
C7	0.105 (9)	0.076 (8)	0.087 (8)	0.014 (7)	−0.009 (6)	−0.012 (6)
C8	0.176 (11)	0.061 (7)	0.140 (10)	0.024 (7)	0.016 (8)	−0.020 (6)
C9	0.049 (3)	0.046 (3)	0.046 (3)	0.000 (3)	−0.004 (2)	0.002 (2)
C10	0.038 (3)	0.042 (3)	0.041 (3)	−0.002 (2)	−0.005 (2)	0.002 (2)
C11	0.043 (3)	0.036 (3)	0.042 (3)	−0.001 (2)	−0.003 (2)	0.002 (2)
C12	0.045 (3)	0.052 (4)	0.043 (3)	0.001 (3)	−0.001 (2)	0.007 (3)
C13	0.046 (3)	0.043 (3)	0.053 (3)	0.001 (2)	−0.011 (2)	0.001 (2)
C14	0.051 (10)	0.048 (10)	0.063 (14)	−0.004 (7)	−0.016 (11)	−0.001 (13)
C15	0.066 (8)	0.050 (10)	0.082 (17)	0.004 (6)	−0.017 (11)	0.016 (11)
C16	0.091 (8)	0.055 (9)	0.143 (11)	0.000 (6)	−0.010 (7)	0.008 (8)
C6'	0.07 (3)	0.07 (2)	0.060 (16)	0.02 (3)	−0.01 (2)	−0.007 (14)
C7'	0.102 (19)	0.072 (17)	0.073 (15)	0.001 (14)	−0.011 (12)	−0.009 (13)
C8'	0.128 (19)	0.101 (17)	0.13 (2)	−0.005 (14)	0.002 (16)	−0.031 (15)
C14'	0.053 (13)	0.047 (12)	0.06 (2)	−0.003 (10)	−0.010 (18)	0.01 (2)
C15'	0.071 (11)	0.051 (12)	0.079 (17)	−0.001 (9)	−0.009 (10)	0.001 (10)
C16'	0.074 (13)	0.09 (3)	0.08 (2)	−0.009 (17)	−0.030 (14)	0.032 (15)

### Geometric parameters (Å, °)

**Table:**

Co1—O1W	2.038 (3)	C5—C6'	1.51 (5)
Co1—O2W	2.083 (3)	C6—C7	1.500 (19)
Co1—N1	2.110 (4)	C6—H6A	0.9700
Co1—N3	2.129 (3)	C6—H6B	0.9700
Co1—O1	2.130 (3)	C7—C8	1.552 (13)
Co1—O5	2.163 (3)	C7—H7A	0.9700
N1—C5	1.319 (5)	C7—H7B	0.9700
N1—C2	1.362 (5)	C8—H8A	0.9600
N2—C5	1.347 (5)	C8—H8B	0.9600
N2—C3	1.361 (5)	C8—H8C	0.9600
N2—H2	0.8600	C9—C10	1.455 (6)
N3—C13	1.323 (5)	C10—C11	1.373 (5)
N3—C10	1.366 (5)	C11—C12	1.473 (6)
N4—C13	1.349 (5)	C13—C14	1.49 (3)
N4—C11	1.357 (5)	C13—C14'	1.52 (6)
N4—H4	0.8600	C14—C15	1.51 (2)
O1—C1	1.261 (5)	C14—H14A	0.9700
O2—C1	1.256 (5)	C14—H14B	0.9700
O3—C4	1.306 (6)	C15—C16	1.52 (2)
O3—H3	0.8200	C15—H15A	0.9700
O7W—C4	1.209 (5)	C15—H15B	0.9700
O5—C9	1.252 (5)	C16—H16A	0.9600
O6—C9	1.271 (5)	C16—H16B	0.9600
O7—C12	1.289 (5)	C16—H16C	0.9600
O7—H7	0.8200	C6'—C7'	1.54 (5)
O8—C12	1.221 (5)	C6'—H6'1	0.9700
O1W—H1W	0.8500	C6'—H6'2	0.9700
O1W—H2W	0.8500	C7'—C8'	1.51 (3)
O2W—H4W	0.8500	C7'—H7'1	0.9700
O2W—H3W	0.8500	C7'—H7'2	0.9700
O3W—H5W	0.8500	C8'—H8'1	0.9600
O3W—H6W	0.8500	C8'—H8'2	0.9600
O4W—H7W	0.8499	C8'—H8'3	0.9600
O4W—H8W	0.8499	C14'—C15'	1.54 (4)
O5W—H9W	0.8499	C14'—H14C	0.9700
O5W—H10W	0.8503	C14'—H14D	0.9700
O6W—H12W	0.8501	C15'—C16'	1.53 (5)
O6W—H11W	0.8503	C15'—H15C	0.9700
C1—C2	1.467 (6)	C15'—H15D	0.9700
C2—C3	1.364 (5)	C16'—H16D	0.9600
C3—C4	1.465 (6)	C16'—H16E	0.9600
C5—C6	1.50 (3)	C16'—H16F	0.9600
			
O1W—Co1—O2W	90.34 (12)	C8—C7—H7B	110.0
O1W—Co1—N1	169.71 (14)	H7A—C7—H7B	108.4
O2W—Co1—N1	88.21 (12)	O5—C9—O6	122.9 (5)
O1W—Co1—N3	89.35 (12)	O5—C9—C10	117.7 (4)
O2W—Co1—N3	170.64 (14)	O6—C9—C10	119.5 (4)
N1—Co1—N3	93.72 (13)	N3—C10—C11	110.0 (4)
O1W—Co1—O1	91.72 (13)	N3—C10—C9	118.4 (4)
O2W—Co1—O1	91.42 (12)	C11—C10—C9	131.6 (4)
N1—Co1—O1	78.13 (13)	N4—C11—C10	105.1 (4)
N3—Co1—O1	97.94 (13)	N4—C11—C12	122.1 (4)
O1W—Co1—O5	89.43 (12)	C10—C11—C12	132.7 (4)
O2W—Co1—O5	93.14 (12)	O8—C12—O7	123.5 (5)
N1—Co1—O5	100.82 (13)	O8—C12—C11	120.6 (5)
N3—Co1—O5	77.50 (13)	O7—C12—C11	116.0 (4)
O1—Co1—O5	175.29 (11)	N3—C13—N4	110.3 (4)
C5—N1—C2	106.3 (4)	N3—C13—C14	126.2 (12)
C5—N1—Co1	142.2 (3)	N4—C13—C14	122.2 (12)
C2—N1—Co1	111.5 (3)	N3—C13—C14'	125.0 (19)
C5—N2—C3	108.6 (4)	N4—C13—C14'	123.3 (19)
C5—N2—H2	125.7	C14—C13—C14'	21.0 (15)
C3—N2—H2	125.7	C13—C14—C15	112 (2)
C13—N3—C10	106.0 (4)	C13—C14—H14A	109.3
C13—N3—Co1	142.2 (3)	C15—C14—H14A	109.3
C10—N3—Co1	111.3 (3)	C13—C14—H14B	109.3
C13—N4—C11	108.7 (4)	C15—C14—H14B	109.3
C13—N4—H4	125.7	H14A—C14—H14B	108.0
C11—N4—H4	125.7	C14—C15—C16	111 (2)
C1—O1—Co1	115.7 (3)	C14—C15—H15A	109.3
C4—O3—H3	109.5	C16—C15—H15A	109.3
C9—O5—Co1	114.9 (3)	C14—C15—H15B	109.3
C12—O7—H7	109.5	C16—C15—H15B	109.3
Co1—O1W—H1W	119.6	H15A—C15—H15B	108.0
Co1—O1W—H2W	129.5	C5—C6'—C7'	105 (2)
H1W—O1W—H2W	108.4	C5—C6'—H6'1	110.7
Co1—O2W—H4W	107.6	C7'—C6'—H6'1	110.7
Co1—O2W—H3W	138.9	C5—C6'—H6'2	110.7
H4W—O2W—H3W	108.3	C7'—C6'—H6'2	110.7
H5W—O3W—H6W	108.6	H6'1—C6'—H6'2	108.8
H7W—O4W—H8W	110.7	C8'—C7'—C6'	114 (3)
H9W—O5W—H10W	109.1	C8'—C7'—H7'1	108.8
H12W—O6W—H11W	106.1	C6'—C7'—H7'1	108.8
O2—C1—O1	124.7 (5)	C8'—C7'—H7'2	108.8
O2—C1—C2	119.0 (4)	C6'—C7'—H7'2	108.8
O1—C1—C2	116.4 (4)	H7'1—C7'—H7'2	107.7
N1—C2—C3	110.0 (4)	C7'—C8'—H8'1	109.5
N1—C2—C1	118.2 (4)	C7'—C8'—H8'2	109.5
C3—C2—C1	131.7 (4)	H8'1—C8'—H8'2	109.5
N2—C3—C2	105.1 (4)	C7'—C8'—H8'3	109.5
N2—C3—C4	121.0 (4)	H8'1—C8'—H8'3	109.5
C2—C3—C4	133.9 (5)	H8'2—C8'—H8'3	109.5
O7W—C4—O3	121.6 (5)	C13—C14'—C15'	110 (2)
O7W—C4—C3	122.0 (5)	C13—C14'—H14C	109.6
O3—C4—C3	116.4 (4)	C15'—C14'—H14C	109.6
N1—C5—N2	110.1 (4)	C13—C14'—H14D	109.6
N1—C5—C6	124.1 (10)	C15'—C14'—H14D	109.6
N2—C5—C6	124.8 (10)	H14C—C14'—H14D	108.1
N1—C5—C6'	127.9 (19)	C16'—C15'—C14'	112 (2)
N2—C5—C6'	119.5 (18)	C16'—C15'—H15C	109.1
C6—C5—C6'	24.0 (15)	C14'—C15'—H15C	109.1
C5—C6—C7	111.0 (14)	C16'—C15'—H15D	109.1
C5—C6—H6A	109.4	C14'—C15'—H15D	109.1
C7—C6—H6A	109.4	H15C—C15'—H15D	107.9
C5—C6—H6B	109.4	C15'—C16'—H16D	109.5
C7—C6—H6B	109.4	C15'—C16'—H16E	109.5
H6A—C6—H6B	108.0	H16D—C16'—H16E	109.5
C6—C7—C8	108.5 (12)	C15'—C16'—H16F	109.5
C6—C7—H7A	110.0	H16D—C16'—H16F	109.5
C8—C7—H7A	110.0	H16E—C16'—H16F	109.5
C6—C7—H7B	110.0		
			
O1W—Co1—N1—C5	165.8 (7)	C2—N1—C5—C6	168.5 (10)
O2W—Co1—N1—C5	83.8 (5)	Co1—N1—C5—C6	−9.7 (12)
N3—Co1—N1—C5	−87.1 (5)	C2—N1—C5—C6'	−162.0 (19)
O1—Co1—N1—C5	175.6 (5)	Co1—N1—C5—C6'	20 (2)
O5—Co1—N1—C5	−9.1 (5)	C3—N2—C5—N1	0.0 (5)
O1W—Co1—N1—C2	−12.3 (9)	C3—N2—C5—C6	−168.8 (9)
O2W—Co1—N1—C2	−94.4 (3)	C3—N2—C5—C6'	163.4 (19)
N3—Co1—N1—C2	94.8 (3)	N1—C5—C6—C7	98.6 (14)
O1—Co1—N1—C2	−2.6 (3)	N2—C5—C6—C7	−94.1 (16)
O5—Co1—N1—C2	172.7 (3)	C6'—C5—C6—C7	−9(5)
O1W—Co1—N3—C13	85.1 (5)	C5—C6—C7—C8	−172.3 (11)
O2W—Co1—N3—C13	173.2 (7)	Co1—O5—C9—O6	−175.8 (3)
N1—Co1—N3—C13	−85.1 (5)	Co1—O5—C9—C10	3.9 (5)
O1—Co1—N3—C13	−6.6 (5)	C13—N3—C10—C11	−0.3 (5)
O5—Co1—N3—C13	174.6 (5)	Co1—N3—C10—C11	173.4 (3)
O1W—Co1—N3—C10	−84.9 (3)	C13—N3—C10—C9	−178.1 (4)
O2W—Co1—N3—C10	3.3 (10)	Co1—N3—C10—C9	−4.5 (5)
N1—Co1—N3—C10	104.9 (3)	O5—C9—C10—N3	0.4 (6)
O1—Co1—N3—C10	−176.5 (3)	O6—C9—C10—N3	−179.9 (4)
O5—Co1—N3—C10	4.7 (3)	O5—C9—C10—C11	−176.8 (4)
O1W—Co1—O1—C1	−178.8 (3)	O6—C9—C10—C11	2.8 (7)
O2W—Co1—O1—C1	90.8 (3)	C13—N4—C11—C10	0.0 (5)
N1—Co1—O1—C1	3.0 (3)	C13—N4—C11—C12	178.7 (4)
N3—Co1—O1—C1	−89.2 (3)	N3—C10—C11—N4	0.2 (5)
O5—Co1—O1—C1	−74.6 (15)	C9—C10—C11—N4	177.6 (4)
O1W—Co1—O5—C9	84.7 (3)	N3—C10—C11—C12	−178.3 (4)
O2W—Co1—O5—C9	175.0 (3)	C9—C10—C11—C12	−0.9 (8)
N1—Co1—O5—C9	−96.2 (3)	N4—C11—C12—O8	−0.6 (6)
N3—Co1—O5—C9	−4.8 (3)	C10—C11—C12—O8	177.6 (4)
O1—Co1—O5—C9	−19.6 (16)	N4—C11—C12—O7	180.0 (4)
Co1—O1—C1—O2	177.2 (3)	C10—C11—C12—O7	−1.8 (7)
Co1—O1—C1—C2	−2.7 (4)	C10—N3—C13—N4	0.2 (5)
C5—N1—C2—C3	0.7 (5)	Co1—N3—C13—N4	−170.0 (3)
Co1—N1—C2—C3	179.5 (3)	C10—N3—C13—C14	−167.0 (10)
C5—N1—C2—C1	−176.7 (4)	Co1—N3—C13—C14	22.7 (12)
Co1—N1—C2—C1	2.1 (4)	C10—N3—C13—C14'	167.2 (15)
O2—C1—C2—N1	−179.6 (4)	Co1—N3—C13—C14'	−3.1 (16)
O1—C1—C2—N1	0.4 (5)	C11—N4—C13—N3	−0.1 (5)
O2—C1—C2—C3	3.8 (7)	C11—N4—C13—C14	167.7 (9)
O1—C1—C2—C3	−176.3 (4)	C11—N4—C13—C14'	−167.3 (15)
C5—N2—C3—C2	0.4 (5)	N3—C13—C14—C15	83 (2)
C5—N2—C3—C4	179.0 (4)	N4—C13—C14—C15	−83 (2)
N1—C2—C3—N2	−0.7 (4)	C14'—C13—C14—C15	178 (10)
C1—C2—C3—N2	176.2 (4)	C13—C14—C15—C16	−172.5 (16)
N1—C2—C3—C4	−179.0 (4)	N1—C5—C6'—C7'	−95 (3)
C1—C2—C3—C4	−2.1 (8)	N2—C5—C6'—C7'	105 (3)
N2—C3—C4—O7W	−0.6 (6)	C6—C5—C6'—C7'	−5(4)
C2—C3—C4—O7W	177.6 (4)	C5—C6'—C7'—C8'	−70 (3)
N2—C3—C4—O3	−179.1 (4)	N3—C13—C14'—C15'	123 (2)
C2—C3—C4—O3	−1.0 (7)	N4—C13—C14'—C15'	−71 (3)
C2—N1—C5—N2	−0.4 (5)	C14—C13—C14'—C15'	23 (6)
Co1—N1—C5—N2	−178.6 (3)	C13—C14'—C15'—C16'	−60 (4)

### Hydrogen-bond geometry (Å, °)

**Table:**

*D*—H···*A*	*D*—H	H···*A*	*D*···*A*	*D*—H···*A*
N2—H2···O4W	0.86	1.89	2.745 (5)	171.
N4—H4···O5W^i^	0.86	1.93	2.752 (5)	160.
O3—H3···O2	0.82	1.68	2.500 (4)	179.
O7—H7···O6	0.82	1.64	2.461 (4)	176.
O1W—H1W···O8^ii^	0.85	1.87	2.715 (4)	178.
O1W—H2W···O3W^iii^	0.85	1.81	2.661 (4)	177.
O2W—H4W···O7W^iv^	0.85	1.94	2.791 (4)	174.
O2W—H3W···O8^v^	0.85	2.05	2.897 (4)	175.
O3W—H5W···O2^iv^	0.85	1.95	2.796 (5)	172.
O3W—H6W···O5^vi^	0.85	2.05	2.895 (4)	172.
O3W—H6W···O6^vi^	0.85	2.63	3.206 (4)	127.
O4W—H8W···O6W	0.85	1.89	2.674 (7)	152.
O5W—H9W···O3W^iii^	0.85	2.08	2.867 (5)	153.
O5W—H10W···O7W^iv^	0.85	2.33	3.092 (5)	149.
O6W—H12W···O6W^vii^	0.85	1.68	2.162 (11)	113.
O6W—H12W···O1^viii^	0.85	2.14	2.730 (6)	126.
O6W—H11W···O5W^iv^	0.85	2.05	2.588 (7)	121.

Symmetry codes: (i) *x*−1, *y*, *z*; (ii) −*x*, −*y*+2, −*z*; (iii) *x*, *y*, *z*−1; (iv) −*x*+1, −*y*+1, −*z*+1; (v) *x*+1, *y*, *z*; (vi) −*x*+1, −*y*+2, −*z*+1; (vii) −*x*+1, −*y*+1, −*z*+2; (viii) *x*, *y*, *z*+1.
